# Circulating Phospholipid Patterns in NAFLD Patients Associated with a Combination of Metabolic Risk Factors

**DOI:** 10.3390/nu10050649

**Published:** 2018-05-21

**Authors:** Shilpa Tiwari-Heckler, Hongying Gan-Schreier, Wolfgang Stremmel, Walee Chamulitrat, Anita Pathil

**Affiliations:** Department of Internal Medicine IV, Gastroenterology and Hepatology, University of Heidelberg, Im Neuenheimer Feld 410, 69120 Heidelberg, Germany; Shilpa.Tiwari-Heckler@med.uni-heidelberg.de (S.T.-H.); Hongying.Gan-Schreier@med.uni-heidelberg.de (H.G.-S.); Wolfgang.Stremmel@med.uni-heidelberg.de (W.S.); Walee.Chamulitrat@med.uni-heidelberg.de (W.C.)

**Keywords:** non-alcoholic fatty liver disease, NAFLD, phospholipids, metabolic disease

## Abstract

Background: Non-alcoholic fatty liver disease (NAFLD) is associated with inefficient macro- and micronutrient metabolism, and alteration of circulating phospholipid compositions defines the signature of NAFLD. This current study aimed to assess the pattern of serum phospholipids in the spectrum of NAFLD, and its related comorbidities and genetic modifications. Methods: 97 patients with diagnosed NAFLD were recruited at a single center during 2013–2016. Based on histological and transient elastography assessment, 69 patients were divided into non-alcoholic steatohepatitis (NASH) and non-alcoholic fatty liver (NAFL) subgroups. 28 patients served as healthy controls. Serum phospholipids were determined by liquid-chromatography mass spectrometry (LC-MS/MS). Results: The total content of phosphatidylcholine (PC) and sphingomyelin in the serum was significantly increased in NAFL and NASH patients, compared to healthy controls. In addition, serum lysophospatidylethanolamine levels were significantly decreased in NAFL and NASH individuals. Circulating PC species, containing linoleic and α-linolenic acids, were markedly increased in NAFLD patients with hypertension, compared to NAFLD patients without hypertension. The pattern of phospholipids did not differ between NAFLD patients with diabetes and those without diabetes. However, NAFLD patients with hyperglycemia (blood glucose level (BGL) >100 mg/dL) exhibited significantly a higher amount of monounsaturated phosphatidylethanolamine than those with low blood glucose levels. In addition, NAFLD patients with proven GG-genotype of PNPLA3, who were at higher risk for the development of progressive disease with fibrosis, showed lower levels of circulating plasmalogens, especially 16:0, compared to those with CC- and CG-allele. Conclusions: Our extended lipidomic study presents a unique metabolic profile of circulating phospholipids associated with the presence of metabolic risk factors or the genetic background of NAFLD patients.

## 1. Introduction

Non-alcoholic fatty liver disease (NAFLD) is one of the most common causes of chronic liver disease in the western world, and is linked to increasing prevalence of obesity and diabetes [1,2]. One of the major contributors to this issue is overconsumption with an inefficient metabolism of macro- and micronutrients, affecting immune response and consequently leading to chronic liver disease [3]. NAFLD extends from lipid accumulation in the liver (non-alcoholic fatty liver (NAFL)) to non-alcoholic steatohepatitis (NASH), which may progress to liver cirrhosis and end-stage liver disease [4,5]. Disturbed hepatic lipid metabolism is a hallmark of NAFLD. Certain lipids, such as diacylglycerol, free fatty acids and ceramides, seem to mediate inflammatory pathways leading to lipotoxicity and oxidative stress, which finally may contribute to disease progression [6]. Previous metabolic studies have provided new insights into altered phospholipid metabolism in NAFLD, implicating an important pathophysiological role for this lipid class [7,8,9]. Puri et al. showed a significant decrease of hepatic phosphatidiylcholine (PC) levels and hepatic lipid subclasses containing arachidonic acid (AA) in NAFLD patients [7]. The progression of NAFL to NASH is characterized by an altered monounsaturated (MUFA) and *n*3- and *n*6-polyunsaturated (PUFA) metabolism, probably due to deranged activities of δ-5, -6, and -9 desaturases, as well as by an impaired PUFA oxidation process, leading to an increase in the total plasma content of non-enzymatic autoxidation products of PUFA lipids, e.g. 11-Hydroxyeicosatetraenoic acid (11-HETE) [7,10], 9-Hydroxyoctadecadienoic acid (9-HODE) and 13-Hydroxyoctadecadienoic acid (13-HODE) [11] or 20-carboxy arachidonic acid (20-COOH AA) [12]. NAFLD is associated with different clinical manifestations of the metabolic syndrome, including obesity, diabetes and hypertension [2,5]. Development of hepatic steatosis and progression to NASH is also linked to genetic background. Genetic association studies recently suggested that the G-allele in patatin-like phospholipase-3 (PNPLA3), which encodes adiponutrin, is strongly associated with hepatic fat content, and more importantly, promotes the susceptibility to develop advanced disease stages with fibrosis [13,14]. Thus, the aim of the present study was to extend lipidomic analysis data on the spectrum of NAFLD, and its associated comorbidities as well as its genetic modifications. Therefore, we performed lipidomic profiling in the serum of healthy controls and patients with NAFLD in order to (1) quantify and compare different circulating phospholipid metabolites; (2) to analyze phospholipids in NAFLD-associated metabolic diseases; and finally to (3) study the association between specific circulating lipids and PNPLA3 polymorphism. 

## 2. Materials and Methods

### 2.1. Study Cohort

97 patients were recruited from the Department of Internal Medicine at the University Hospital of Heidelberg from 2013–2016. 28 patients served as healthy controls. These control patients had normal liver enzyme levels and did not have any record of liver disease, and therefore showed no indication for liver biopsy. All individuals had routine clinical and hematological examinations. NAFLD was suspected by the presence of elevated liver enzymes and after exclusion of other causes of liver disease (e.g., viral or autoimmune hepatitis, M. Wilson, hemochromatosis, significant alcohol use). In addition, ultrasound examinations and transient elastography with FibroScan^®^ 502 Touch (Echosens™) were conducted. Liver biopsy was performed in 41 patients. The diagnosis of NASH was based on the following criteria: (1) Elevated liver enzymes (AST > 46 U/L, ALT > 50 U/L); (2) hepatic steatosis on ultrasound; and (3) histological examination or (4) elevated Fibroscan results (>7 kPa). 42 patients were assigned to the NASH cohort according to these criteria. 25 patients were included in the NAFL group: 5 patients because of liver histology results and 20 patients who exhibited only one or two of the above mentioned criteria. Blood samples of the 97 patients were taken and immediately prepared, and stored at −80 °C for further analysis, as shown below. Genotype of the PNPLA3 rs738409 gene of 52 patients was investigated after patient informed consent. The institutional review board approved the study. 

### 2.2. Profiling of Phospholipids and Sphingolipids

The patterns of phospholipids and sphingolipids were determined by using XBridge C18 Column from Waters, Milford, MA, USA and HPLC-ESI/MSMS (triple-quadrupole Micro Mass Quattro 81 Premier, Waters, Milford, MA, USA, coupled with HTC Pal auto sampler, CTC Analytics, Zwingen, Switzerland), as described in Jiao et al. [15]. In brief, a constant amount of internal standards, containing 17:0 lysophosphatidylcholine (lysoPC), 14:0/14:0 phosphatidylcholine (PC), 12:0/12:0 phosphatidylethanolamine (PE), and 17:0 Ceramide (Cer), were added to each serum sample. A classical Folch method, which uses chloroform/methanol (3:2, *v*/*v*), was applied to the glass tubes [16]. After evaporation, lipid extracts were resolved in methanol and were analyzed using HPLC-ESI/MSMS. For the purpose of data acquisition and processing, Masslynx version 4.1 software (Waters, Milford, MA, USA) was used.

### 2.3. Data Analysis

Data are presented as mean ± SEM and were analyzed with GraphPad Prism 5.0 (GraphPad Software, La Jolla, CA, USA). Groups were compared using unpaired two-tailed students’ *t*-test. One-way ANOVA, including Bonferroni post-hoc test, was used for comparing three or more groups. A *p*-value less than 0.05 was considered statistically significant.

## 3. Results

### 3.1. Baseline Characteristics

The study population included 69 patients, who presented at the Department of Internal Medicine at the University Hospital of Heidelberg with the suspected diagnosis of NAFLD. 28 patients served as healthy controls. All patients underwent clinical examination, laboratory data were collected and additionally, ultrasound examinations and transient elastography were performed. Forty-one patients underwent liver biopsy. According to the histological assessment, 5 patients were diagnosed with NAFL and 34 patients were grouped as NASH patients. Two patients were excluded from the study, as they had no pathological findings indicating NAFLD. Based on Fibroscan values (F2–F4), further 8 patients were added to the NASH group. 20 patients were included in the NAFL cohort, as they presented with Fibroscan values under F2. NASH (*n* = 42) and NAFL (*n* = 25) patients had significantly higher levels of liver enzymes, triglycerides and total cholesterol in comparison to healthy controls (*n* = 28) (Table 1). 

### 3.2. Increase of Circulating PC and Sphingomyelin and Decrease of Phosphatidylethanolamine in NAFL and NASH

**Figure 1:**
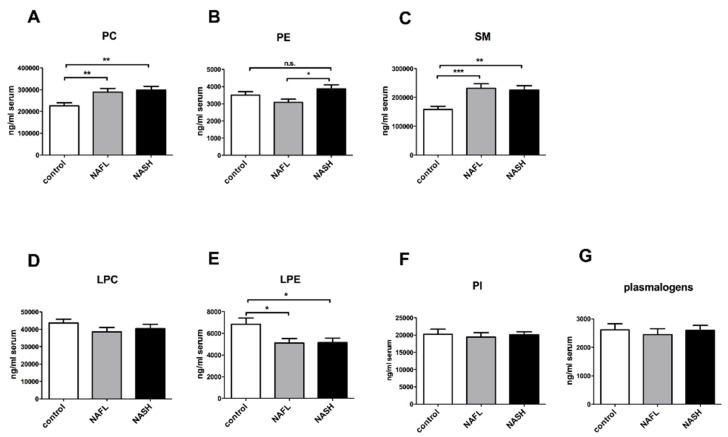
Circulating phospholipids in control (*n* = 28), NAFL (*n* = 25) and NASH (*n* = 42) cohorts. Results of lipid profiling of 95 patients by using LC-MS/MS method. Patients were divided in NAFL and NASH cohorts. 28 patients served as healthy control. Quantification of (**A**) PC; (**B**) PE; (**C**) SM; (**D**) LPC; (**E**) LPE; (**F**) PI; and (**G**) plasmalogens in the serum of healthy, NAFL and NASH patients in ng/mL serum. All the values are presented in mean ± SEM. * *p* < 0.05, ** *p* < 0.01, *** *p* < 0.001, n.s. = not significant (*p* > 0.05).

By using LC-MS/MS, we identified and quantified 140 species of phospholipids and sphingomyelin (SM) in the serum. The total content of PC and SM in the serum was significantly increased in NAFL and NASH patients, compared to healthy controls (Figure 1A,C). Contrary to PC, the level of lysophosphatidylcholine (LPC) showed a trend to be decreased in both the NAFL and NASH groups, without reaching significance (Figure 1D). Circulating phosphatidylethanolamine (PE) was elevated in patients with NASH, compared to those with NAFL (Figure 1B). The amount of lysophosphatidylethanolamine (LPE) was significantly decreased in NAFL and NASH patients in comparison to controls (Figure 1E). The level of phosphatidylinositol (PI) and plasmalogens did not differ between all groups (Figure 1F,G).

### 3.3. Altered Phospholipid Composition in NASH Patients with Metabolic Risk Factors

**Figure 2:**
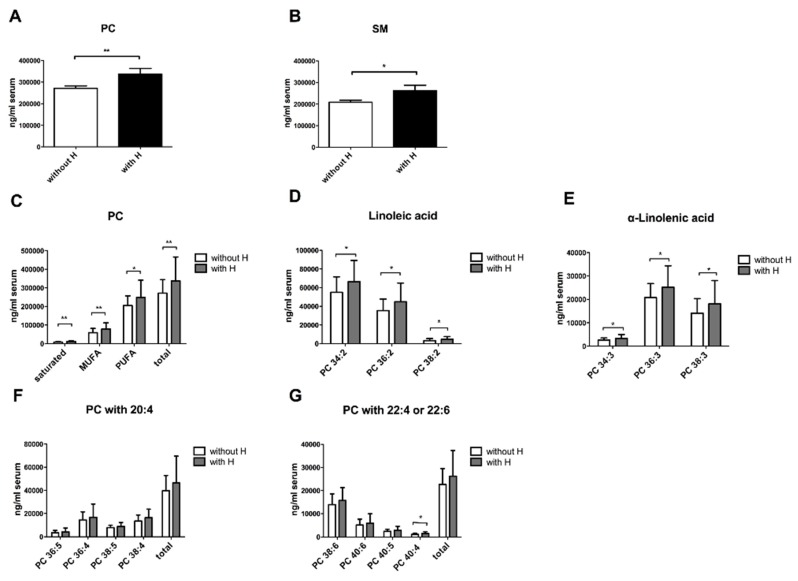
Overview of circulating phospholipids in NAFLD patients with and without hypertension. NAFLD patients were divided into patients with hypertension (with H; *n* = 24) and without hypertension (without H; *n* = 43). (**A**) Quantification of circulating PC and SM is represented in ng/mL serum. (**B**) Distribution of PC subtypes, namely saturated, mono- and poly-unsaturated PC levels, are displayed in these two groups in ng/mL serum. The total content of PC is evaluated as a sum of saturated and unsaturated PC. (**C**–**E**) Circulating PC, containing linoleic acids (PC 34:2, PC 36:2, PC 38:2) and α-linolenic acids (PC 34:3, PC 36:3, PC 38:3), are compared between hypertensive and normotensive NAFLD patients. (**F**,**G**) The amount of arachidonic acid (PC 20:4, 22:4 and 22:6) in PC is demonstrated in this graphic. All the values are presented in mean ± SEM. * *p* < 0.05, ** *p* < 0.01.

**Figure 3:**
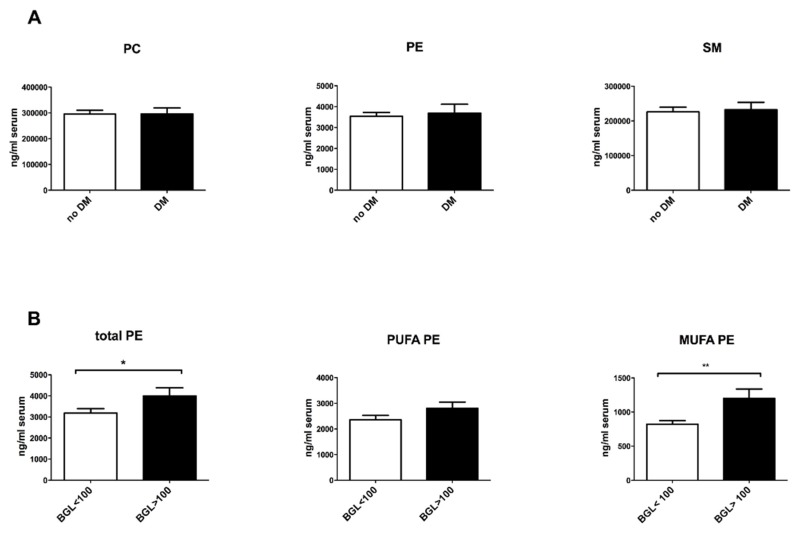
MUFA-PE is increased in NAFLD patients with hyperglycemia. (**A**) Quantification of circulating PC, PE and SM in ng/mL serum in NAFLD patients who were divided into patients with diabetes mellitus (DM; *n* = 18) and without diabetes mellitus (no DM; *n* = 49). (**B**) Quantification of total PE, PUFA-PE and MUFA-PE in ng/mL serum in NAFLD patients with a BGL over 100 mg/dL (BGL > 100; *n* = 16) or under 100 mg/dL (BGL < 100; *n* = 22). All the values are presented in mean ± SEM. * *p* < 0.05, ** *p* < 0.01.

We investigated the contribution of phospholipid species in NAFLD-associated comorbidities. NAFL and NASH patients were enrolled in groups regarding the presence of diabetes, hypertension and obesity. Interestingly, NAFLD patients with hypertension exhibited significantly higher levels of PC and SM (Figure 2A,B) in the serum than NAFLD patients without hypertension. Circulating PC species, containing linoleic acids (PC 34:2, PC 36:2, PC 38:2) and α-linolenic acids (PC 34:3, PC 36:3, PC 38:3), were markedly increased in these patients, whereas those containing arachidonic acid remained unaffected (Figure 2D–G). The sum of phospholipids, including PC, PE and SM, did not differ between diabetes (*n* = 18) and non-diabetes NAFLD patients (*n* = 49) (Figure 3A). However, further analysis revealed a correlation between MUFA-PE and hyperglycemia within the NAFLD cohort. 16 patients with a blood glucose level (BGL) over 100 mg/dL showed higher levels of serum PE, which was due to a significant elevation of MUFA-PE, whereas PUFA-PE remained unchanged (Figure 3B). Obese NAFLD patients (body mass index (BMI) > 30 kg/m^2^) showed no significant differences, compared to healthy (BMI < 25 kg/m^2^) and overweight (BMI 25–30 kg/m^2^) NAFLD patients (Supplementary Materials Figure S1). 

### 3.4. Reduction of Circulating Plasmalogens in Patients with PNPLA3 GG-Genotype

**Figure 4:**
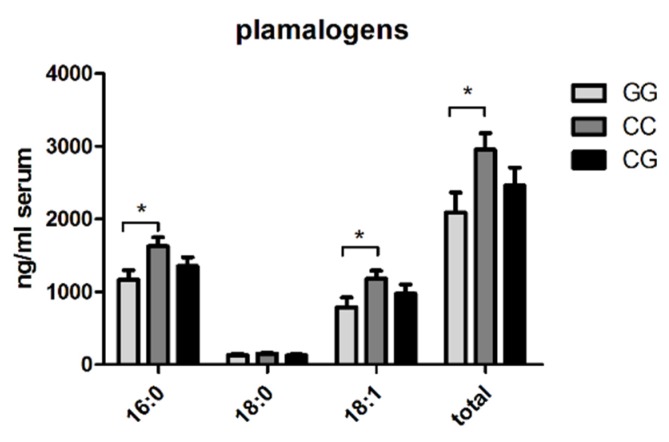
Association between plasmalogens and PNPLA3 gene polymorphism. The PNPLA3 rs738409 genotype was determined in 52 NAFLD patients. The total level of plasmalogen was the sum of plasmalogen 16:0, 18:0 and 18:1. The amount of these plasmalogen subtypes was quantified in NAFLD patients with GG- (*n* = 14), CC- (*n* = 20) and CG-allele (*n* = 18) of PNPLA3. Circulating plasmalogen subclasses are displayed in ng/mL serum. All the values are presented in mean ± SEM. * *p* < 0.05.

Genetic analysis regarding the determination of the PNPLA3 rs738409 genotype was performed in 52 NAFLD patients. Lipid profiling of these patients showed lower levels of total plasmalogens, especially 16:0 and 18:1, in patients with PNPLA3 GG-genotype (*n* = 14), compared to those with CC- (*n* = 20) or CG- (*n* = 18) allele (Figure 4). 

## 4. Discussion

It is well known that NAFLD is the hepatic manifestation of the metabolic syndrome [1]. Our extended lipidomic analysis provides an insight into the complex changes in circulating lipid patterns and lipid levels related to NAFLD-associated metabolic risk factors, as well as to the individual genetic background, potentially provoking metabolic and immunomodulatory responses in liver. 

It has been recently discovered that altered PC metabolism is involved in the progression of human NAFL to NASH [7]. We also confirm hereby that the content of circulating PC is increased in NAFL and NASH patients, compared to healthy controls. We could not measure a significant change in the PC product, i.e., LPC in our cohorts. One possible explanation is the further utilization of LPC, e.g., by autotaxin. It has been recently reported that plasma autotaxin levels are increased in different chronic liver diseases, including NASH [17]. Total PE content was elevated in NASH patients, compared to NAFL in our study. However, data on PE analysis were inconsistent. Puri et al. and Wattacheril et al. showed that PE levels seemed to be unchanged in NASH compared to healthy liver [7,9], but as recently presented by Ma et al., accumulation of PE could be associated with disease progression [18]. 

However, we discovered that LPE might serve as a novel biomarker for NAFL and NASH, as this phospholipid subgroup was significantly reduced in the NAFLD cohort. We suggest that the lack of LPE and consequently the inability to further metabolize LPE to PC, can lead to disease progression. To compensate the decrease of hepatocellular PC, therapeutic approaches, including PC supplementation by food intake, were conducted [19]. Stremmel et al. showed a protective and anti-inflammatory role of topical PC in chronic ulcerative colitis [20]. However, the treatment of liver diseases with PC was not as successful in humans as in mouse models, because a replenishment of high PC levels in hepatocytes was not easily achievable [21,22]. As direct application of PC is of no therapeutic benefit, pro-drugs, serving as PC precursors, have been developed [23,24]. For instance, our group synthetized a drug candidate, which consists of the bile acid (UDCA) and the PC precursor LPE [25], and which owns hepatoprotective and anti-inflammatory functions in a high fat diet mouse model of NAFLD by changing hepatic fatty acid composition [24,26]. 

Our LC-MS/MS analyses revealed that circulating PC, containing linoleic (LA) and α-linolenic acids (ALA), was increased in NAFLD patients with hypertension. With changing nutritional behavior over the last few decades, western diet is now characterized by high fat diet and suboptimal micronutrient intake [3]. In particular, intake and metabolism of essential fatty acids are relevant to the pathogenesis of different chronic diseases [27,28]. Essential PUFAs, which are not produced by the human body, include LA and ALA [19,29]. Few epidemiological studies suggest a positive impact of dietary intake of essential PUFAs on cholesterol profiling or blood pressure [19,30]. However, these associations seem to be inconsistent in the literature, as other investigations imply the opposite effect or do not recognize any difference [27,31]. Additionally, an excess of LA can lead to adverse effects because of an increase of oxidized LA metabolites [29]. For instance, oxidized LDL compounds derive from LA and contribute to atherosclerosis [32]. In vitro studies demonstrate pro-inflammatory properties of LA, during liver injury by stimulating endoplasmatic reticulum stress and hepatocyte apoptosis and by increasing pro-inflammatory cytokines, such as TNFα, which leads to Kupffer cell activation [33,34]. Accordingly, in clinical settings, oxidized derivates of LA, such as 9- and 13-HODE, are correlated with NASH [11]. LA and ALA can also be converted to arachidonic acids [29], which remained unaltered in our cohorts. Our data indicate a positive association between circulating essential fatty acids and NAFLD-associated hypertension.

Although we did not discover lipid alterations by comparing diabetes and non-diabetes NAFLD patients, further analysis regarding BGLs revealed an increase of PE, especially MUFA-PE, in NAFLD patients with a BGL over 100 mg/dL. Glycation of PE could lead to an Amadori-linked product, which could induce lipid peroxidation. The level of Amadori-PE was increased in diabetic patients and was considered as a possible marker of a hyperglycemic condition, particularly in the early stage of diabetes [35,36]. 

In our next step, our lipid profiling in the NAFLD group identified a decrease of plasmalogens in patients with the GG-genotype of PNPLA3, compared to those with the CC- and CG-genotypes. 

Recent studies highlighted a strong association between a variant (rs738409 C>G p. I148M) in the *PNPLA3* gene and steatosis, fibrosis and the development of hepatocellular carcinoma in multiple chronic liver diseases, including metabolic disorders, alcoholic liver disease and chronic viral hepatitis. The I148M substitution represents a loss of function mutant that contributes to triglyceride accumulation in hepatocytes, which may lead to increased lipid peroxidation and oxidative stress (lipotoxicity) [37,38]. A recent study by Carpino et al. analyzed serum from NAFLD patients with I148M variant, showing high levels of serum systemic oxidative stress markers, such as F2-isoprostanes and Nox2 activity [37]. 

Plasmalogens are synthesized in peroxisomes and prevent cellular damage induced by oxidative stress, and thus, seem to have anti-inflammatory functions [39,40]. As recently published by Jang and colleagues, endogenous hepatic plasmalogens appear to be involved in fatty acid metabolism and inhibit steatosis and NASH progression through PPARα-dependent signaling [41]. It is known that oxidative stress-induced peroxisome dysfunction limits plasmalogen biosynthesis, and thus, attenuates the release of plasmalogens to the blood [39]. Puri et al. reported low circulating serum plasmalogen levels in patients presenting with NASH in their lipidomics study [10].

Interestingly, we discovered a positive association of PNPLA3 I148M carriers and low levels of circulating plasmalogens, compared to patients with CC- or CG-genotype. This observation suggests a possible pathogenetic link on the cellular level, underlining the association between triglyceride accumulation, oxidative stress, peroxisome dysfunction and accordingly, defects of plasmologens biosynthesis. Moreover, their loss of function may aggravate NASH progression. This molecular mechanism between PNPLA3 I148M mutant and plasmologens still remains unclear and needs to be elucidated in further experimental and clinical studies.

In recent publications, the most frequently used categorization of NAFLD patients in NASH and NAFL was due to histological assessment. In our cohort study, 28 patients denied liver biopsy in spite of medical recommendation. Ultrasound examination and transient elastography with Fibroscan were performed in all sessions. Therefore, 39 patients could be divided in NAFL and NASH subgroups based on histological analyses. Eight patients exhibited liver stiffness >F2 by transient elastography and were included to the NASH group. This kind of categorization may lead to sample selection bias. However, lipid profiling in 39 patients with only histological assessment revealed a significant increase of high levels of PC and SM and a marked decrease of LPE in the NASH cohort (34 patients), compared to healthy controls, consistent with the lipid analysis in our groups with results of histology and transient elastography. No significant deviation could be detected between NAFL and control patients. It should be noted that only 5 individuals from 39 patients with histological analyses could be assigned to the NAFL group (Supplementary Materials Figure S2).

## 5. Conclusions

In summary, this lipidomic profiling investigated circulating phospholipid metabolites in NAFLD patients. This current study confirmed that PC is significantly increased in the serum of NASH patients and is identified as a possible novel biomarker in the pathogenesis of NAFLD, namely LPE. Our analysis elucidated lipidomic alterations in NAFLD patients with hypertension, demonstrating a key role for PC species containing essential fatty acids in NAFLD-associated hypertension. Furthermore, metabolic profiling in this NAFLD cohort determined a correlation between high levels of MUFA-PE and hyperglycemia. Finally, we discovered a link between low levels of circulating plasmalogens and NAFLD patients with the unfavorable GG genotype of PNPLA3.

## Figures and Tables

**Table 1:** Baseline characteristics and laboratory results from healthy controls, NAFL and NASH patients.

	Control (*n* = 28)	NAFL (*n* = 25)	NASH (*n* = 42)
Age (years)	39.4 ± 2.6	46.4 ± 2.9	43.7 ± 2.2
Sex (M/F)	13/15	14/11	29/13
AST (U/L)	20.7 ± 1.3	43.6 ± 5.8 ***	55.3 ± 6.0 ***
ALT (U/L)	22.0 ± 1.7	68.9 ± 7.9 ***	86.3 ± 9.5 ***
GGT (U/L)	21.8 ± 2.4	144.4 ± 24.6 ***	218.1 ± 49.6 **
Bilirubin (mg/dL)	0.6 ± 0.04	0.9 ± 0.2	1.4 ± 0.6
Triglycerides (mg/dL)	83.4 ± 5.9	152.2 ± 12.6 ***	187.3 ± 14.6 ***
Total cholesterol (mg/dL)	182.2 ± 9.0	213.2 ± 7.8 *	169.3 ± 12.2
HDL cholesterol (mg/dL)	54.7 ± 3.9	53.1 ± 3.7	46.7 ± 2.2
LDL cholesterol (mg/dL)	110.8 ± 6.4	128.2 ± 6.6	113.3 ± 6.2

M = Male, F = Female, AST = Aspartate aminotransaminase, ALT = Alanine aminotransaminase, GGT = Gamma-glutamyl transpeptidase, HDL = High-density lipoproteins, LDL = Low-density lipoprotein. Values are represented in mean ± SEM. *p*-values vs. control. * *p*-value < 0.05, ** *p*-value < 0.01, *** *p*-value < 0.001.

## Supplementary Materials

The following are available online at http://www.mdpi.com/2072-6643/10/5/649/s1. Figure S1: Comparison of phospholipid subclasses in NAFLD-associated obesity. Figure S2: Quantification of PL species in healthy controls (*n* = 28), NAFL (*n* = 5) and NASH (*n* = 34) cohorts.
