# Supplementary material for: Circulating Phospholipid Patterns in NAFLD Patients Associated with a Combination of Metabolic Risk Factors
Source: Nutrients. 2018 May 21;10(5):649. doi: 10.3390/nu10050649 (PMC5986528; doi:10.3390/nu10050649)
Supplement: Supplementary file 1 [file nutrients-10-00649-s001.pdf]

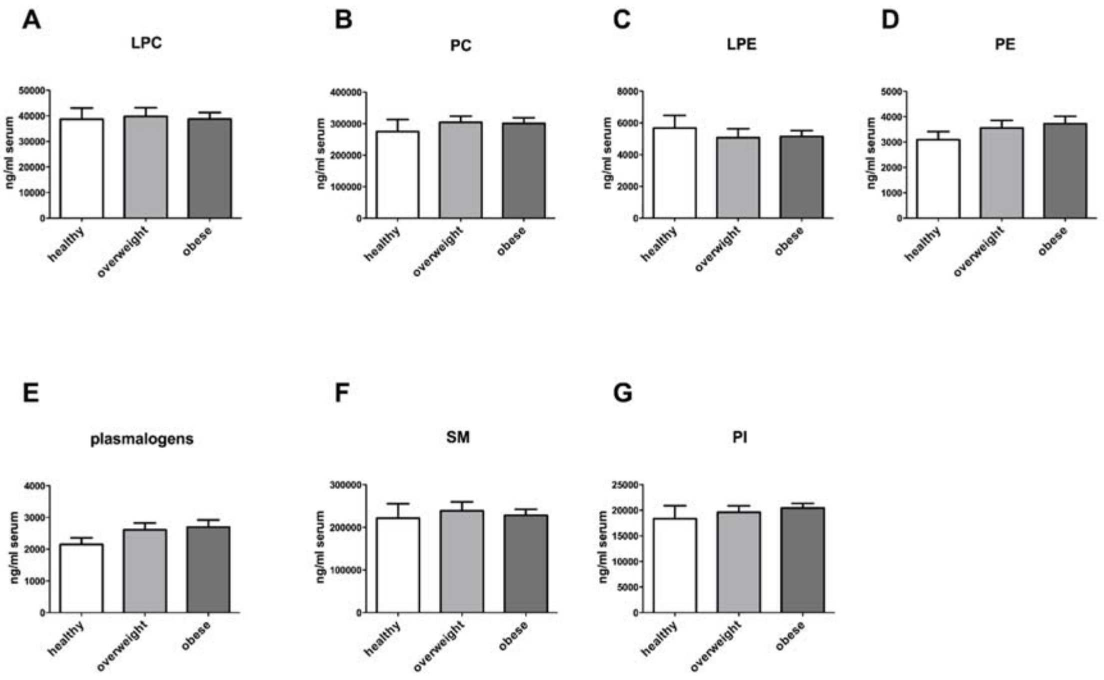

Supplementary Materials Figure S1. Comparison of phospholipid subclasses in NAFLD-associated obesity. NAFLD patients were divided in three groups based on their BMI values: obese patients with BMI > 30 kg/m² included 28 patients. Patients with BMI value 25–30 kg/m² (n = 27) were considered as overweight. 8 patients had a BMI < 25 kg/m² and were regarded as healthy.

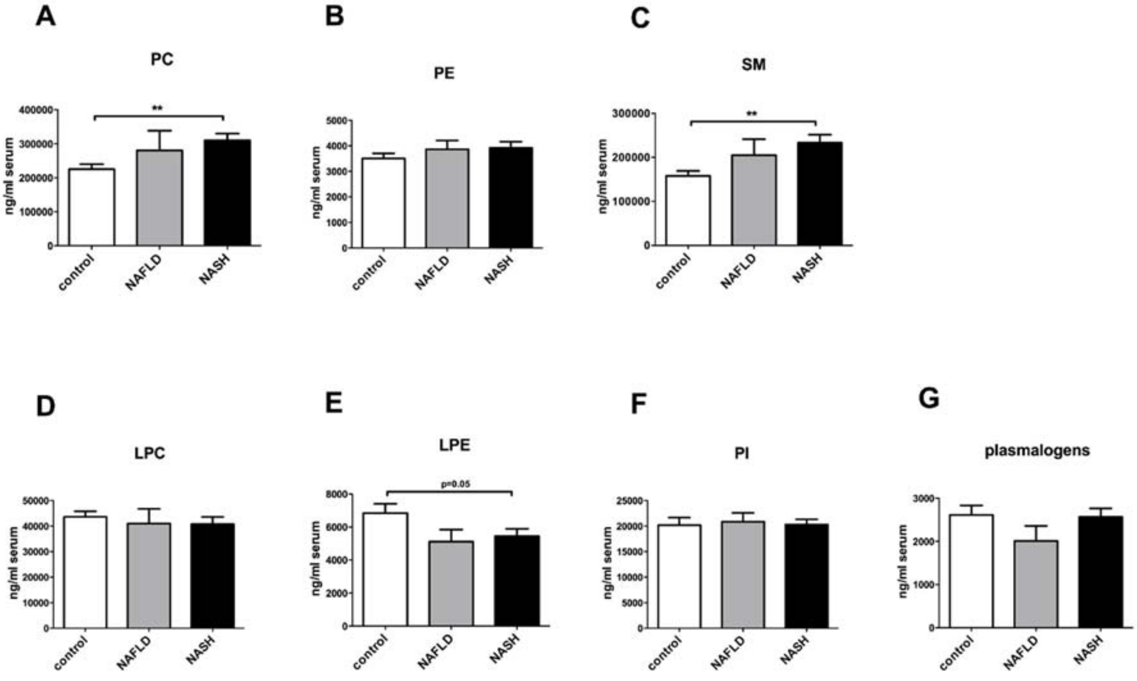

Supplementary Materials Figure S2. Quantification of PL species in healthy controls (n = 28), NAFL (n = 5) and NASH (n = 34) cohorts. Patients were grouped in NAFL and NASH cohorts based on the histological results of their liver biopsies (A-D) Lipid levels in these groups were represented in bars (x- axis) in ng/mL serum (y- axis). All values are presented in mean ± SEM. * $p < 0.05$, ** $p < 0.01$.
